# Coulomb force-guided deep reinforcement learning for effective and explainable robotic motion planning

**DOI:** 10.3389/frobt.2025.1697155

**Published:** 2026-01-30

**Authors:** Sirui Song, Trevor Bihl, Jundong Liu

**Affiliations:** Learning and Intelligent Systems Lab (LiSL), School of Electrical Engineering and Computer Science, Ohio University, Athens, OH, United States

**Keywords:** Coulomb force, deep reinforcement learning, Gazebo, lidar, motion planning, TurtleBot3

## Abstract

Training mobile robots through digital twins with deep reinforcement learning (DRL) has gained increasing attention to ensure efficient and safe navigation in complex environments. In this paper, we propose a novel physics-inspired DRL framework that achieves both effective and explainable motion planning. We represent the robot, destination, and obstacles as electrical charges and model their interactions using Coulomb forces. These forces are incorporated into the reward function, providing both attractive and repulsive signals to guide robot behavior. In addition, obstacle boundaries extracted from LiDAR segmentation are integrated as anticipatory rewards, allowing the robot to avoid collisions from a distance. The proposed model is first trained in Gazebo simulation environments and subsequently deployed on a real TurtleBot v3 robot. Extensive experiments in both simulation and real-world scenarios demonstrate the effectiveness of the proposed framework. Results show that our method significantly reduces collisions, maintains safe distances from obstacles, and generates safer trajectories toward the destinations.

## 1 Introduction

LiDAR-based mobile robot navigation marks a significant advancement in robotics, offering a wide range of advantages and applications. Unlike traditional global-map-based systems, LiDAR generates a real-time, detailed 3D map of the robot’s surroundings, enabling operators to make informed decisions with precise spatial data. This capability is crucial in dynamic and unpredictable environments, where adaptive, sensor-driven awareness outperforms reliance on fixed perspectives.

Motion planning and collision avoidance are critical components of high-performance robotic autonomy. Traditional motion planning approaches typically rely on geometric, sampling-based, or optimization-based methods to create feasible and efficient paths from a starting point to a goal while avoiding obstacles. Graph-based methods, such as the A* (Hart et al., 1968), and D* (Stentz, 1995) alongside sampling-based techniques like Rapidly-Exploring Random Trees* (RRT*) (Karaman and Frazzoli, 2011) and Probabilistic Roadmap (PRM) (Yang et al., 2018), remain among the most widely adopted solutions.

In recent years, machine learning (ML)-based solutions have gained popularity for enabling mobile robots to perceive their environments and make maneuvering decisions. Supervised learning methods perform perception and decision-making simultaneously, directly predicting control policies from sensor data such as images (Kim and Chen, 2015; Giusti et al., 2015; Tai et al., 2016; Dai et al., 2020; Back et al., 2020) and LiDAR scans (Chen et al., 2020; Murillo, 2023). In contrast, reinforcement learning (RL) (Michels et al., 2005) allows robots to learn optimal navigation strategies through trial and error. By interacting with the environment and receiving feedback, robots can gradually enhance their navigation performance. When combined with neural networks, deep reinforcement learning (DRL) has demonstrated superhuman performance in various games (Mnih et al., 2015; Xie et al., 2017; He et al., 2020). More recently, DRL-based solutions for collision avoidance and goal-reaching have also been proposed (Singla et al., 2019; Xue and Gonsalves, 2021; Song et al., 2022; Chang et al., 2021; Ouahouah et al., 2021; Olayemi et al., 2023). To reduce costs and improve effectiveness, training is often initially conducted in simulated environments.

LiDAR-based DRL methods have been investigated in recent studies, with particular attention to intrinsic motivation as a means to improve generalization. Zhelo et al. (2018) addressed RL limitations in scenarios such as long corridors and dead ends by incorporating an intrinsic curiosity module, which enhanced exploration and outperformed predefined reward functions in virtual 2D tasks (Mirowski et al., 2016; Long et al., 2018). Shi et al. (2019) applied a similar curiosity-driven approach within an A3C framework using sparse LiDAR input, enabling policies to transfer effectively from simulation to realistic mixed environments.

In parallel, researchers have explored novel architectural designs to address persistent challenges in motion planning. Wang et al. (2018) decomposed planning into obstacle avoidance and goal navigation, employing raw laser rangefinder data within a dual-stream Q-network to generate force-based actions. Kim et al. (2021) proposed a DQN-GRU-based navigation method that incorporated action skipping to improve performance in partially observable MDP-modeled environments, achieving superior results in simulation compared to standard DQN and non-skipping baselines. To address cross-task generalization, Wang et al. (2020) introduced elastic weight consolidation (EWC) into a DDPG framework, enabling policies to preserve prior knowledge and mitigate catastrophic forgetting without full retraining. Yan et al. (2023) also employed DDPG for mapless navigation and demonstrated improved performance in unknown environments compared to A*.

While DRL-based LiDAR navigation methods show considerable promise, their architectures are often constrained by reward designs that lack strong physical grounding. In particular, the reward structures in existing approaches frequently lack clear physical interpretation. For instance, several studies (Xue et al., 2019; Gao et al., 2020; Song et al., 2021) employ a fixed distance penalty, yet its actual impact remains insufficiently examined. Furthermore, many classical path-planning techniques, such as artificial potential fields, have not been effectively integrated into DRL frameworks. Finally, approaches remain limited to simulated validation, and even those tested on real-world robots often provide little quantitative evidence to confirm their effectiveness in practical deployment.

In this paper, we propose a physics-inspired DRL-based motion planning algorithm that generates continuous commands without relying on a map. We utilize Coulomb force to model interactions between the robot, its destination, and surrounding obstacles. To enhance safety, we incorporate object segmentation, enabling the robot to anticipate and avoid collisions from a distance. A 2D LiDAR sensor provides the data necessary to support the robot’s behaviors, including collision avoidance and goal-reaching. We also develop a carefully designed method to enhance the generalization of our solution, validated across various environment settings.

The proposed designs are trained and tested in Gazebo simulation scenes (Robotics, 2014) with large geometric obstacles and deployed in real TurtleBot v3 (TB3) robots (Robotics, 2017). Experiments conducted in both simulated and real environments demonstrate the effectiveness of our overall design and individual components.

The contributions of our work can be summarized as follows:

- We model the robot, the destination, and obstacles as electrical charges and use Coulomb forces to model the interactions between these charges.
- These Coulomb forces are integrated into our DRL framework, providing encouraging and preventative rewards for the robots. The proposed Coulomb-based rewards are smooth and pervasive, offering consistent guidance throughout the entire training field. To the best of our knowledge, this is the first work ever to employ Coulomb force in path planning and reinforcement learning.
- Obstacle boundaries extracted from LiDAR segmentation enable the robot to anticipate and avoid collisions from a distance.
- The proposed Coulomb- and vision-based rewards have clear, interpretable effects on robot behavior and performance, thereby providing strong overall explainability of our model.
- We carefully design environment-invariant components in our DRL system to improve the generalization of our solution.

## 2 Background

### 2.1 Gravitational and coulomb forces

Gravity, as formulated by Isaac Newton, is a force of attraction that acts between all objects with mass. Mathematically, it is expressed as

$$F_{\text{gravity}}=G\frac{m_{1}m_{2}}{r^{2}},$$

where $m_{1}$ and $m_{2}$ are the masses of the two objects, r is the distance between their centers, and G is the universal gravitational constant. This relationship indicates that the gravitational force grows larger with increasing mass and weaker with increasing distance, following an inverse-square law.

**FIGURE 1:**
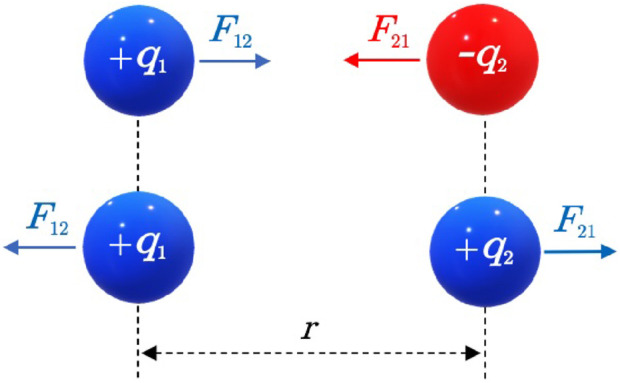
Coulomb force between two particles $q_{1}$ and $q_{2}$ can be either attractive or repulsive depending on the signs of the charges.

Coulomb’s force, in contrast, describes the electrostatic interaction between charged particles. As illustrated in Figure 1, Coulomb’s law quantifies the strength of the repulsive or attractive force between two point charges, such as a proton and an electron in an atom. The law states that the electric force exerted by one charge on another depends on the magnitudes of the charges and the square of the distance r between them. In its simplest form, Coulomb’s law for the magnitude of this force is expressed as:

$$F_{12}=F_{21}=k_{e}\frac{\left|q_{1}q_{2}\right|}{r^{2}},$$ (1)

where $q_{1}$ and $q_{2}$ are electric charges, r is the distance between the charges, and $k_{e}$ is the Coulomb constant. This force can be attractive when $q_{1}$ and $q_{2}$ have opposite signs or repulsive when $q_{1}$ and $q_{2}$ have the same sign. Like gravity, it follows an inverse-square dependence on distance.

The two forces share a striking mathematical similarity, both decreasing with the square of the distance between interacting entities. In fact, the forms of Newton’s law of gravitation and Coulomb’s law look very much alike, reflecting the inverse-square nature common to both. Nonetheless, they differ fundamentally in that gravity is always attractive, while electrostatic forces can attract or repel. Another key difference is their relative strength: on atomic and subatomic scales, the electrostatic force between charged particles is far stronger than their mutual gravitational attraction. Yet over astronomical distances, neutrality of charge means that gravity dominates, shaping the large-scale structure of the universe by pulling together planets, stars, and galaxies into stable orbits and clusters.

### 2.2 Classical and RL-Based motion planning methods

Robot motion planning methods can be broadly divided into classical and RL-based approaches. Classical planners follow a structured pipeline of global planning, local obstacle avoidance, and trajectory generation, often using graph search or sampling strategies such as A*, RRT*, or DWA (Fox et al., 2002). While reliable in static, well-mapped environments, they struggle in dynamic or uncertain settings due to rigid rule-based logic and the need for labor-intensive map construction. Early classical methods were limited to simple static models (Philippsen and Siegwart, 2003; Cosío and Castañeda, 2004) or treated dynamic objects as static at discrete time steps (Borenstein and Koren, 1990; Borenstein and Koren, 1991), restricting real-world applicability. More recent efforts have improved collision avoidance through algorithmic refinements, including A* and DWA for real-time navigation (Kherudkar et al., 2024), GBI-RRT combined with SLAM (Sun et al., 2023), and enhanced DWA variants for dynamic environments (Cao and Nor, 2024).

In contrast, RL-based motion planning can be broadly categorized into hybrid, end-to-end, and multi-robot approaches. Hybrid methods integrate RL with classical planners to combine reliability with adaptability, such as DRL-enhanced DWA for smoother trajectories (Wang and Huang, 2022), A2C with optimization outperforming Dijkstra + DWA (Xing et al., 2022), and DRL combined with A* to reduce computation (Liu et al., 2024). These methods, however, remain dependent on map quality and consistency. End-to-end methods learn directly from sensor data, with LiDAR-based DQN-GRU achieving superior performance over standard DQN (Kim et al., 2021), stochastic sampling with 2D LiDAR enabling faster training and improved collision avoidance (Beomsoo et al., 2021), and LSTM-TD3 models offering improved temporal decision-making (Wen et al., 2024).

While promising for handling unseen environments, RL-based solutions suffer from high training demands and limited real-world generalization. Multi-robot approaches often adopt centralized training and decentralized execution (CTDE), such as enhanced DQN for warehouse path planning a DRL-MPC-GNN framework for task allocation and coordination (Li et al., 2024), and curriculum-learning with LiDAR costmaps yielding strong real-world results (Yu et al., 2024). Memory-augmented DQN variants have also improved multi-robot coordination (Quraishi et al., 2025). Despite scalability and coordination benefits, these methods face challenges in training complexity, dynamic interactions, and partial observability, limiting their practical deployment in robot systems.

### 2.3 Artificial potential field (APF) algorithm

The APF algorithm, first proposed by Khatib (Khatib, 1986), is a classical physics-inspired framework for real-time path planning and obstacle avoidance. In this approach, the robot is modeled as a particle moving under the influence of a synthetic potential function U(x) that combines attractive and repulsive components:

$$U\left(x\right)=U_{\text{att}}\left(x\right)+U_{\text{rep}}\left(x\right),\;F\left(x\right)=-\nabla U\left(x\right)$$

where F(x) represents the virtual force acting on the robot.

The attractive potential pulls the robot toward its goal and is commonly modeled as a quadratic function of the Euclidean distance:

$$U_{\text{att}}\left(x\right)=\frac{1}{2}k_{\text{att}}\|x-x_{g}{\|}^{2},\;F_{\text{att}}\left(x\right)=-k_{\text{att}}\left(x-x_{g}\right)$$

where $k_{\text{att}}>0$ controls the rate of attraction, x is the robot’s current location and $x_{g}$ denotes the goal position.

The repulsive potential is activated only within a finite influence range $d_{0}$ to maintain a safety margin from obstacles:

$$U_{\text{rep}}\left(x\right)=\left\{\begin{array}{cc} \frac{1}{2}k_{\text{rep}}{\left(\frac{1}{d\left(x\right)}-\frac{1}{d_{0}}\right)}^{2},\; & d\left(x\right)\leq d_{0},\\ 0,\; & d\left(x\right)>d_{0}, \end{array}\right.\;F_{\text{rep}}\left(x\right)=-\nabla U_{\text{rep}}\left(x\right),$$

where $k_{\text{rep}}>0$ is a scaling factor, d(x) is the distance from robot to obstacle, and $d_{0}$ defines the repulsion boundary.

The resultant virtual force $F\left(x\right)=F_{\text{att}}\left(x\right)+F_{\text{rep}}\left(x\right)$ guides the robot toward the goal while avoiding obstacles. Despite its simplicity and intuitive physical interpretation, APF suffers from two major limitations: (1) the handcrafted potential shapes and distance thresholds make the field non-convex and highly sensitive to parameter tuning, and (2) the method is prone to local minima in concave or cluttered environments where attractive and repulsive forces may cancel, preventing progress toward the goal.

The DRL framework presented in this work is motivated by the principles of the APF algorithm. To overcome its inherent limitations, we design RL reward functions that yield a physically grounded and globally smooth motion-guidance field. This field is realized using Coulomb-force–based rewards, with the full formulation presented in Section 3.

### 2.4 Generalization and sim-to-real transfer in DRL

Model generalization is a critical issue in machine learning, and it is especially important for DRL-based navigation and control. In robotics, the ability of a policy to adapt to new or changing environments is vital, as operating conditions are often unpredictable and diverse. A well-generalized policy can handle unseen scenarios, task variations, and sensor differences, whereas a poorly generalized one risks catastrophic failure outside its training distribution. Despite its importance, many DRL studies still evaluate methods only on the same environments they were trained on, such as Atari (Bellemare et al., 2013), Gazebo (Koenig and Howard, 2004), or OpenAI Gym (Brockman et al., 2016), providing limited insight into generalization.

Recent efforts have begun to address this issue. Yu et al. (Yu et al., 2020) studied the generalization of multiple DRL algorithms by training across diverse environments, while Doukui et al. (Doukhi and Lee, 2021) mapped sensor data, robot states, and goals to continuous velocity commands, though their work was restricted to unseen targets rather than unseen scenes. Increasingly, DRL-based robot obstacle avoidance research has emphasized sim-to-real transfer in (Lee et al., 2022; Wu et al., 2023; Joshi et al., 2024). For instance, Anderson et al. (Anderson et al., 2021) introduced a subgoal model that aligns simulation-trained discrete actions with real-world continuous control, using domain randomization to reduce visual discrepancies. Similarly, Zhang et al. (Zhang et al., 2021) applied object detection to generate real-time 3D bounding boxes, mitigating the effect of varying obstacle shapes and appearances on robot navigation and improving robustness to sim-to-real differences.

## 3 Methods

In this work, a mobile robot begins at a specified location and autonomously navigates toward a target destination. Static and dynamic obstacles are placed along the straight line connecting the start and end points. The primary objective is to enable the robot to reach the destination while effectively avoiding collisions. This capability is achieved through a physics-inspired DRL framework with two key considerations: (1) obstacle avoidance and (2) generalization to previously unseen environments.

The proposed models are first trained in the Gazebo simulation environment and then deployed on a TB3, which functions as a simple two-wheeled differential-drive robot. The TB3 is equipped with multiple sensors, including a 360° LiDAR and an Inertial Measurement Unit (IMU). It also supports the Robot Operating System (ROS 2), which enables seamless communication and control between the DRL algorithms and the hardware platform.

**FIGURE 2:**
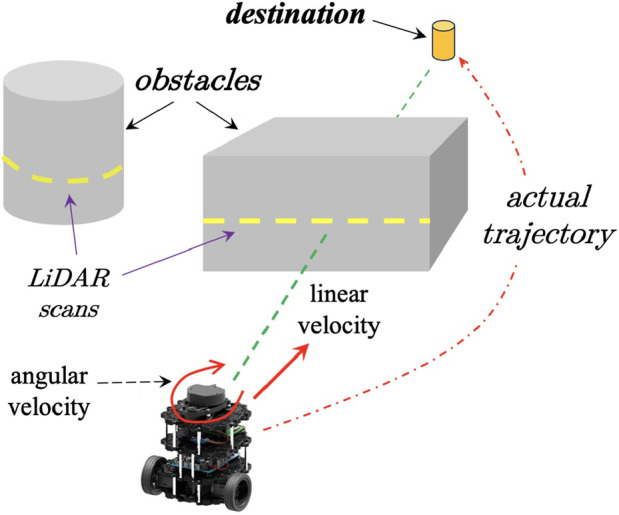
An illustration of the overall motion planning setup. Refer to text for details.

Figure 2 illustrates the experimental setup. The green dashed line represents the straight path between the robot’s initial position and the destination, while the red dashed line shows the actual trajectory, deviating from the path to avoid obstacles. At each time step, the LiDAR produces a distance map of sampled points from the surrounding environment, represented by yellow dashed lines on the obstacles. The robot’s motion is controlled by two velocity components: (1) linear velocity, which determines the speed of forward movement, and (2) angular velocity, which controls the rotation rate of the two-wheeled base.

### 3.1 Overall design and key innovations

The primary innovation of this work lies in: (1) modeling the robot, destination, and obstacles as charged particles, and (2) utilizing Coulomb forces to represent their interactions while formulating DRL rewards based on these interactions. Specifically, the robot and its destination are modeled as electric charges with opposite signs, generating an attractive force that guides the robot toward the goal. Obstacles are represented as an array of charges with the same sign as the robot, producing a combined repulsive force that steers the robot away from collisions. In both cases, the force magnitudes scale inversely with the square of the distance between interacting entities.

Our design introduces two breakthrough innovations that enable highly effective agent learning. The first is the use of gradually varying and ubiquitous forces, which provide consistent guidance across the entire training field. The second is the inverse-square distance formulation, which is particularly effective for collision avoidance, as the repulsive force increases sharply when the robot approaches an obstacle. By incorporating the robot’s direction of movement, these forces are translated into reward signals for the DRL agent, either encouraging or redirecting its trajectory.

Our innovative designs have clear physical interpretations, making the behavior and performance of our model highly explainable. Furthermore, we propose an object-avoidance reward based on LiDAR scan segmentation, which enables the robot to avoid large obstacles from a distance, significantly enhancing the overall performance of the models.

**FIGURE 3:**
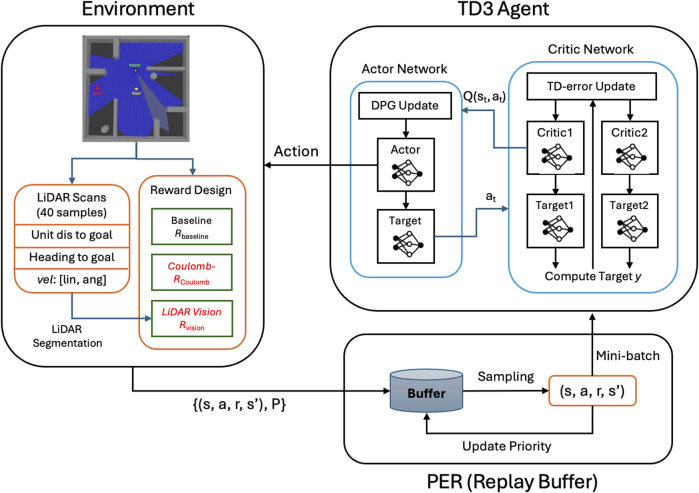
Overview of the proposed Coulomb-guided TD3 framework with LiDAR-based perception.

The overall workflow of the proposed framework is illustrated in Figure 3, which outlines the interaction among the environment, a Twin Delayed Deep Deterministic Policy Gradient (TD3) control agent (Fujimoto et al., 2018), and Prioritized Experience Replay (PER) (Schaul et al., 2016). The environment interacts with the agent through continuous sensing and motion feedback. The proposed rewards are generated directly from these interactions and serves as the primary learning signals for the agent. A standard TD3 algorithm is employed as the training backbone to validate the effectiveness and generality of the proposed reward formulation, while PER is applied to improve sample efficiency during training.

This framework emphasizes the role of the proposed reward, which provides dense and physically interpretable feedback directly from environment interactions. The integration of Coulomb-force and LiDAR-vision rewards significantly improves learning stability and convergence speed compared with conventional sparse rewards. Experimental analysis further demonstrates reduced collision frequency and enhanced path planning capability, confirming that the proposed reward design improves both the efficiency and robustness of policy learning. These observed improvements in convergence speed, stability, and trajectory efficiency directly reflect the effectiveness of the proposed reward formulation, which operationalizes the main research contributions within the defined DRL framework.

### 3.2 DRL algorithm selection

The TD3 algorithm was chosen as the primary DRL framework due to its inherent stability, high sample efficiency, and robustness in continuous control tasks. TD3 extends the Deep Deterministic Policy Gradient (DDPG) (Lillicrap et al., 2015) method by introducing twin Q-networks to mitigate overestimation bias and delayed policy updates to prevent divergence, resulting in smoother convergence. Such properties are particularly important for velocity-based robot navigation, where unstable value estimation can lead to oscillatory motion or unsafe control.

Although Proximal Policy Optimization (PPO) (Schulman et al., 2017) has been successfully applied to various robotic control and path planning problems, its on-policy nature requires frequent policy rollouts and gradient updates, leading to lower sample efficiency and higher computational cost in long-horizon navigation tasks. In contrast, TD3’s off-policy structure enables efficient experience reuse from replay buffers, accelerating convergence while maintaining stable policy updates.

Meanwhile, Soft Actor–Critic (SAC) (Haarnoja et al., 2018) enhances exploration through entropy regularization, which is advantageous in sparse-reward or high-uncertainty environments. However, in our Coulomb-guided setup, where the reward function already provides strong directional gradients and the control space demands consistent velocity regulation, the additional stochasticity of SAC’s policy can introduce unnecessary action variance and make policy updates less stable.

As a result, a deterministic actor-critic algorithm such as TD3 offers a more direct and stable optimization path for continuous navigation tasks with dense, physics-guided rewards, aligning well with the objectives of this work.

### 3.3 States and actions

States, actions, and rewards are the three fundamental components of most DRL algorithms. In this work, the state at time t , denoted $s_{t}$ , is defined as:

$$s_{t}=[{\text{dist}}_{\mathrm{obst}}\left(t\right),\;{\text{unit\_dist}}_{\mathrm{goal}}\left(t\right),\;{\text{angle}}_{\mathrm{goal}}\left(t\right),\;\text{lin\_speed}\left(t\right),\;\text{ang\_speed}\left(t\right)]$$

where:

- LiDAR-based distance map $\left({\text{dist}}_{\mathrm{obst}}\left(t\right)\right)$ : obtained from 40 LiDAR samples (9-degree each). The minimum value in the scan is extracted at each step, reflecting the likelihood of collision.
- Unit distance to the goal $\left({\text{unit\_dist}}_{\mathrm{goal}}\left(t\right)\right)$ : defined as the ratio of the current distance to the goal over the maximum (initial) distance, serving as a normalized proximity measure.
- Goal angle $\left({\text{angle}}_{\mathrm{goal}}\left(t\right)\right)$ : the angular difference between the robot’s current heading and the destination direction.
- Robot velocities: linear velocity lin_speed(t) and angular velocity ang_speed(t) , where angular velocity is expressed in degrees per second clockwise.

The design of these state components is intended to enhance generalization. The LiDAR-derived shortest distance captures proximity to obstacles in an environment-independent manner. The goal angle ${\text{angle}}_{\mathrm{goal}}\left(t\right)$ generates a scale- and environment-independent rotational factor, while the unit distance ${\text{unit\_dist}}_{\mathrm{goal}}\left(t\right)$ both drives forward motion and quantifies progress toward the destination.

At each step, the action $a_{t}$ is represented as:

$$a_{t}=\left[\text{lin\_speed}\left(t+1\right),\;\text{ang\_speed}\left(t+1\right)\right]$$

which updates the robot’s linear and angular velocities from their values at time t . The design of the reward functions, derived from these states and actions, will be detailed in the next subsection.

### 3.4 Reward design

In this work, the overall reward $r_{t}$ at time t is designed to incorporate multiple components, each corresponding to a desired system behavior. The design goals include: (1) encouraging the robot to reach the destination, (2) avoiding collisions, and (3) enhancing model generalization. The overall reward is expressed in Equation 2,

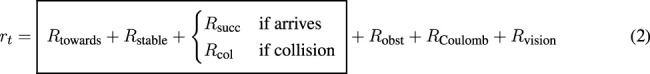

where:

-
$R_{\text{towards}}$ : incentivizes the robot to move closer to the target,
-
$R_{\text{stable}}$ : rewards smooth and stable motion by minimizing excessive rotation and acceleration,
-
$R_{\text{succ}}$ : provides a significant positive reward when the robot successfully reaches its destination,
-
$R_{\text{col}}$ : imposes a penalty (negative reward) if the robot collides with obstacles,
-
$R_{\text{obst}}$ : an interval-based function that penalizes the robot when it gets too close to obstacles,
-
$R_{\text{Coulomb}}$ : combines attractive and repulsive terms derived from Coulomb forces, guiding the robot toward the goal while repelling it from obstacles,
-
$R_{\text{vision}}$ : allows the robot to see upfront from a distance and encourages the robot to move toward regions free of collisions.

The four (4) reward terms in the box of Equation 2 represent the baseline rewards, indicating that these terms are included in the reward function of every model.

Among these the baseline rewards, $R_{\text{towards}}$ can be formulated in different ways. In this study, we define it as the sum of two components: (1) a reward for reducing ${\text{angle}}_{\mathrm{goal}}\left(t\right)$ , which encourages the robot to align its heading with the target, and (2) a reward for reducing the normalized distance to the destination ${\text{unit\_dist}}_{\mathrm{goal}}\left(t\right)$ . These two components, shown in Equation 3, work together to effectively incentivize progress toward the goal:

$$R_{\text{towards}}=-c_{1}\cdot |{\text{angle}}_{\mathrm{goal}}|+\frac{2\times {\text{dist}}_{\mathrm{goal-ini}}}{{\text{dist}}_{\mathrm{goal-ini}}+{\text{dist}}_{\mathrm{goal}}}$$ (3)

where $c_{1}$ is a positive scaling coefficient, ${\text{dist}}_{\mathrm{goal-ini}}$ is the initial distance to the goal, and ${\text{dist}}_{\mathrm{goal}}$ is the current distance.

$R_{\text{stable}}$ is designed to ensure that the robot moves smoothly and steadily, avoiding sudden turns or unstable oscillations. To achieve this, the stable reward consists of two components: (1) a penalty on the rotational angular velocity ang_speed(t) , particularly when it is excessively high, and (2) a penalty on the linear velocity lin_speed(t) when the robot moves too slowly, i.e., when its velocity is significantly lower than the maximum robot speed, Max_Speed . In this work, the TB3 used in real-world experiments has a maximum speed of 0.22 m per second. The stable reward is defined as:

$$R_{\text{stable}}=-c_{21}\cdot \text{ang\_speed}{\left(t\right)}^{2}-c_{22}\cdot {\left(Max\text{\_}Speed-\text{lin\_speed}\left(t\right)\right)}^{2}$$

where $c_{21}$ and $c_{22}$ are positive constants.

$R_{\text{obst}}$ is designed to repel the robot when it gets too close to obstacles. This is implemented by applying a constant penalty whenever the minimum distance between the robot and the nearest obstacle falls below a predefined threshold. In our setup, the threshold is set to 0.22 m, corresponding to the distance the robot would travel in one second at its maximum speed, which could otherwise result in a collision. Formally, $R_{\text{obst}}$ is defined as:

$$R_{\text{obst}}=\left\{\begin{array}{cc} -20,\; & \text{if }\min\left[{\text{dist}}_{\mathrm{obst}}\left(t\right)\right]\leq \text{threshold}\\ 0,\; & \text{otherwise} \end{array}\right.$$

$R_{\text{Coulomb}}$ and $R_{\text{vision}}$ are reward components designed to guide the robot’s movement. Specifically, $R_{\text{Coulomb}}$ drives the robot toward the goal while simultaneously repelling it from nearby obstacles, and $R_{\text{vision}}$ encourages the robot to avoid obstacles from a greater distance. Both rewards will be explained in detail in the following subsections.

#### 3.4.1 Coulomb force rewards *R*_Coulomb_

As previously introduced, we model the robot, destination, and obstacles as charged particles and utilize Coulomb’s law to represent their interactions. These interactions form the basis of the Coulomb-based reward, $R_{\text{Coulomb}}$ , which consists of two components: an attractive force reward from the goal that pulls the robot toward the destination, and a repulsive force reward from obstacles that pushes the robot away to avoid collisions. The overall formulation is given by:

$$R_{\text{Coulomb}}=R_{\text{attr}}+R_{\text{repu}}$$

Attraction Reward $R_{\text{attr}}$ is designed to attract the robot to the destination as quickly as possible. In this model, the robot is represented as a positive charge and the destination as a negative charge. By Coulomb’s law, oppositely charged particles attract each other, and the attractive force between the robot and the destination is calculated accordingly. The closer the robot is to the destination, the stronger this attraction becomes.

**FIGURE 4:**
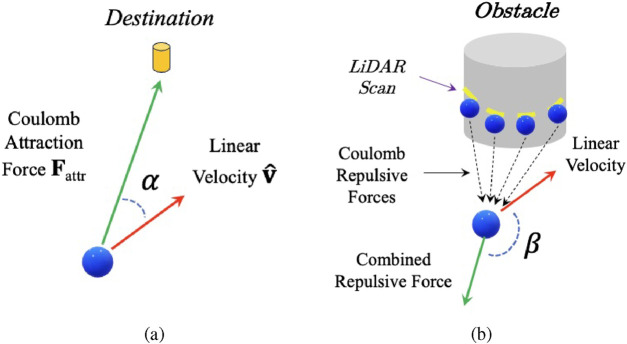
An illustration of the Coulomb force rewards $R_{\text{Coulomb}}$ setup. **(a)** Attractive force from the destination; **(b)** repulsive forces from obstacle points. Refer to text for details.

Figure 4a illustrates the attractive force exerted by the destination on the robot. To map this attraction into a reward for the DRL agent, we compute the inner product between the attractive force vector $F_{\text{attr}}$ and the robot’s direction of motion (linear velocity $\hat{v}$ ). This inner product serves as the attraction reward, encouraging the robot to align heading direction towards the destination as closely as possible. Inspired by Coulomb’s law, $R_{\text{attr}}$ is formulated as:

$$R_{\text{attr}}=F_{\text{attr}}\cdot \hat{v}=\|F_{\text{attr}}\|\cdot \cos\left({\text{angle}}_{\mathrm{goal}}\right)=c_{3}\cdot \frac{1}{{\text{dist}}_{\mathrm{goal}}^{2}}\cdot \cos\left({\text{angle}}_{\mathrm{goal}}\right)$$

where $c_{3}$ is a positive constant, ${\text{angle}}_{\mathrm{goal}}$ is the angle between the force $F_{\text{attr}}$ and robot’s motion direction $\hat{v}$ , and ${\text{dist}}_{\mathrm{goal}}$ is the distance between the robot and the destination. This relationship is illustrated in Figure 4a.

$R_{\text{repu}}$ is designed to keep the robot away from obstacles. As the robot approaches an obstacle, a repulsive force is generated to push it away, thereby preventing collisions. We model the robot as a positively charged particle with a charge of $q_{1}=1$ (see Figure 4b). Each LiDAR scan point is likewise modeled as a positively charged particle with a charge of $q_{2}=1$ . According to Coulomb’s law, these like-charged particles exert repulsive forces on one another, as expressed in Equation 1. By summing the individual repulsive force vectors, we obtain a resultant force that prevents the robot from moving closer to obstacles, as illustrated in Figure 4b.

This resultant force is then used to compute the Coulomb-based repulsive reward applied to the robot. For simplicity, Coulomb’s constant is set to 1. The final reward function for the repulsive force is defined as:

$$R_{\text{repu}}=-c_{4}\cdot \overset{N}{\underset{i=1}{\sum }}\frac{1}{d_{i}^{2}}\cdot \cos\left(\theta_{i}\right)$$

where $c_{4}$ is a positive constant; $d_{i}$ is the distance between the robot and the i -th obstacle point (from LiDAR), and $\theta_{i}$ is the angle between the i -th repulsive force vector and the robot’s direction of motion. In this study, N is set to 40.

Our Coulomb-force–based reward was originally inspired by the classical APF formulation, both of which use a physics-inspired combination of attractive and repulsive influences to guide robot motion. In this sense, our formulation conceptually extends the idea of shaping a force field that directs the robot toward the goal while avoiding obstacles. But method, however, has several key differences and advancements over APF:

- Algorithmic vs. Learning-Based Mechanism The classical APF method is algorithmic and deterministic, meaning its behavior remains fixed and does not refine or improve with repeated experience. In contrast, our Coulomb-force rewards are integrated into a learning-based DRL framework. The agent receives reward signals from its local neighborhood and continuously improves its policy through interaction and extensive training. This training process allows the agent to discover effective behavioral patterns that reflect the underlying physical field, leading to more sophisticated navigation and improved escape from local minima where traditional APF typically becomes trapped.
- Smooth, Globally Consistent Reward Field The reward formulation we propose produces a smooth, ubiquitous, and gradually varying guidance field. This contrasts with classical APF, where handcrafted potential shapes and discontinuous distance thresholds often create non-convex fields prone to sharp gradients and local minima. The smoothness and physical consistency of the Coulomb-based reward help the learned policy achieve more stable motion guidance and reduce susceptibility to local minima.
- Exploration and Stochasticity in DRL Our learning model, as a DRL approach, possesses an exploration capability which acts analogously to a simulated annealing process. This property helps the agent occasionally deviate from local optima and, consequently, discover more globally efficient paths.

#### 3.4.2 Vision rewards *R*_vision_

**FIGURE 5:**
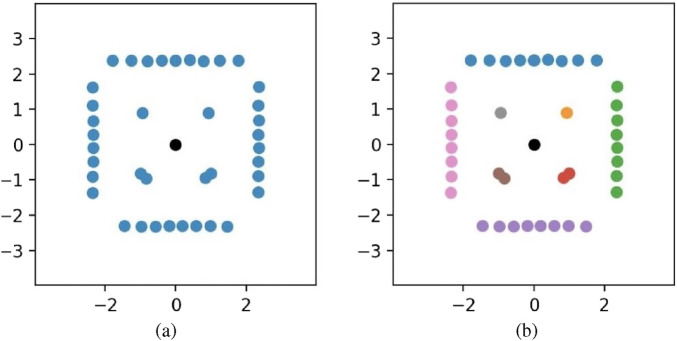
Example of LiDAR segmentation. **(a)** shows the original LiDAR scan with 40 samples; **(b)** displays the corresponding segmentation result after applying DIET, where different colors represent distinct objects.

$R_{\text{vision}}$ encourages the robot to move tangentially along obstacle edges within collision-free zones, enabling early avoidance and improving both the success rate and efficiency of obstacle evasion. To identify the boundary of the object ahead, we first perform LiDAR scan segmentation on all scan points. By classifying these points, we can distinguish individual objects, as illustrated in Figure 5, which shows the segmentation result using 40-sample LiDAR scans. From the classified points, we select the object directly facing the robot and determine its leftmost and rightmost LiDAR points. The corresponding angles with respect to the robot’s heading direction are denoted as $\theta_{1}$ and $\theta_{2}$ . The smaller of the two angles is chosen to minimize the effort required for obstacle avoidance, and a fixed offset is applied to this angle to maintain a safety buffer.

Since obstacle avoidance is less urgent when obstacles are farther away, we normalize the reward using a Gaussian function of the potential collision time $t_{c}$ . In this formulation, imminent collisions generate stronger reward signals, whereas distant collisions contribute weaker signals. This design ensures that the reward varies smoothly with decreasing collision time, resulting in smoother trajectories and more efficient obstacle avoidance.

**FIGURE 6:**
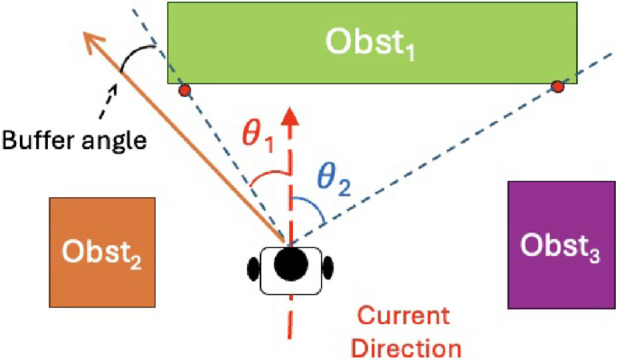
An illustration of the LiDAR vision reward setup. Refer to text for details.

Formally, the vision reward is defined as:

$$R_{\text{vision}}=c_{5}\cdot \cos\left(\min\left(\theta_{1},\theta_{2}\right)+\text{buffer}\right)\cdot \exp\left(-\frac{t_{c}^{2}}{2\sigma^{2}}\right),$$

where $c_{5}$ is a positive constant, σ is set to 1, $\theta_{1}$ and $\theta_{2}$ denote the angles between the robot’s orientation and the lines connecting the robot to the leftmost and rightmost boundaries of the object ahead (with a fixed safety buffer, as shown in Figure 6), and $t_{c}$ is the estimated collision time, calculated from the obstacle distance and the robot’s speed.

**FIGURE 7:**
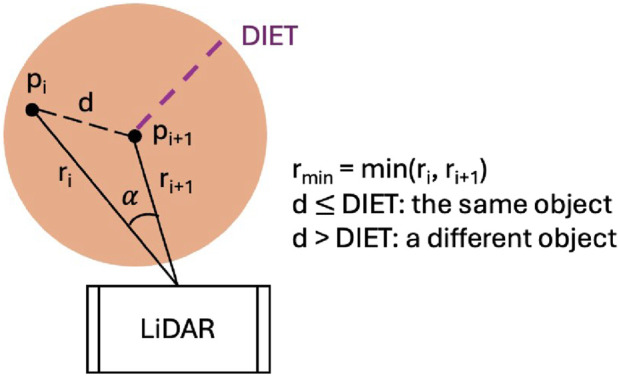
Illustration of the DIet algorithm applied to LiDAR scan segmentation.

In more detail, we employ the DIET (Dietmayer, 2001) algorithm for LiDAR segmentation. The procedure, illustrated in Figure 7, operates by examining the distances between adjacent LiDAR scan points to identify potential object boundaries. This allows neighboring points that belong to the same physical surface to be grouped together, thereby enabling effective segmentation of obstacles. The DIET function is defined as:

$$DIET=C_{0}+C_{1}\cdot \min\left(r_{i},r_{i+1}\right)$$

where $C_{0}$ is a positive constant used to reduce noise; $C_{1}$ is a positive constant dependent on the angle α between two LiDAR beams; $r_{i}$ and $r_{i+1}$ denote the distances from two adjacent scan points to the robot’s position (0, 0).

## 4 Environment and model setups

We design training and testing environments in Gazebo to simulate the TB3 Burger’s motion planning and collision avoidance under realistic conditions. After training and validating the models in simulation, we deploy them on the TB3 to evaluate their performance in real-world scenarios. Training is conducted on an NVIDIA RTX A6000 GPU. In this framework, simulation serves as the primary stage for model development and verification, while real-world experiments provide the final assessment of robustness and reliability.

### 4.1 Simulation environments

**FIGURE 8:**
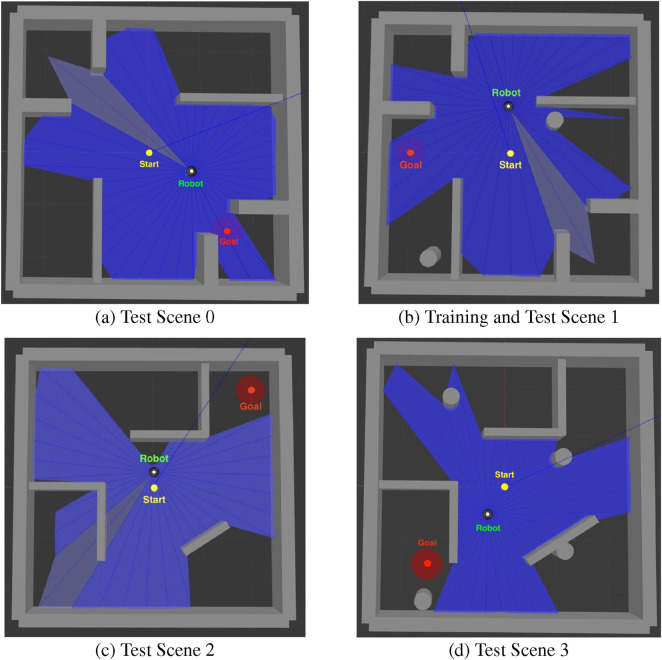
Simulation environments for model training and testing. **(a)** Test scene 0 without moving obstacles; **(b)** training and test scene 1 with moving obstacles; **(c)** test scene 2 without moving obstacles; and **(d)** test scene 3 with moving obstacles. Gray cylinders in **(b,d)** denote dynamic (moving) obstacles.

The motion planning policies are trained in Gazebo using a digital twin of the TB3 robot provided by the manufacturer. The simulation environments, illustrated in Figure 8, include both training and testing setups. Figures 8a,b (referred to as Scene 0 and Scene 1) depict the same environment, with 8(b) containing additional moving obstacles. The DRL model is trained in 8(b), Scene 1, since it represents a more complex environment. Figures 8c,d (Scene 2 and Scene 3) present an unseen environment used exclusively for testing, designed to evaluate the model’s generalization and robustness. In 8(a) and 8(c), only static obstacles (walls) are present, while 8(b) and 8(d) include dynamic obstacles represented by gray cylinders.

In all environments, the robot starts at the center of the scene (0,0). At the beginning of each epoch, a goal is randomly selected from a predefined set of locations. If the robot reaches the goal without collision, it continues toward a new goal from its current position. In the event of a collision, the environment is reset by relocating the robot to the center and assigning a new random goal.

### 4.2 Real environments

**FIGURE 9:**
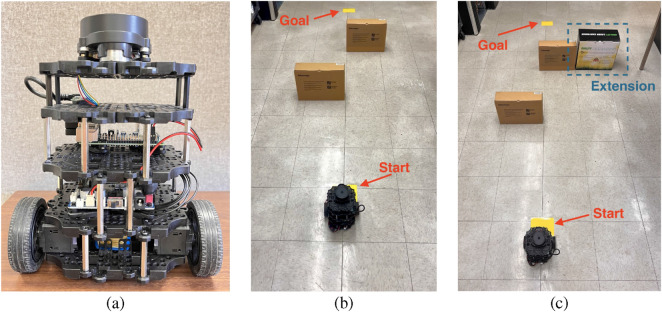
Deployment of a TB3 in real-world environments. **(a)** Our robot; **(b)** Real Test Scene 1; **(c)** Real Test Scene 2: with an extended obstacle.

In the real-world experiments, we used the TB3 to evaluate our DRL-based models. The hardware setup is shown in Figure 9a. From top to bottom, the robot is equipped with a 360° LiDAR for scan acquisition, a Raspberry Pi 4 for wireless communication over WiFi, an OpenCR board that controls the robot and exchanges data with the Raspberry Pi via USB, and a LiPo battery at the base that powers the entire system. The DRL models are executed on a remote PC, with action values transmitted to the Raspberry Pi 4 in real time through ROS 2.

Sim-to-real transfer remains a major challenge for RL-based algorithms, as models that perform well in simulation may fail in physical environments. Therefore, real-world testing is essential. Figure 9b shows the first real-world test environment (referred to as Real Test Scene 1), which contains two obstacles to evaluate each model’s collision avoidance and goal-reaching capabilities. Figure 9c depicts a second test environment (Real Test Scene 2), where one obstacle is extended to further test the robot’s ability to find alternative paths. The third test environment, not shown in the figure, builds on the first by introducing dynamic obstacles, allowing assessment of the robot’s performance under moving hazards. Success is defined as reaching within a radius of r=0.3 m from the goal at coordinates (3,0). In all real-world tests, the robot starts at (0,0), as indicated in Figures 9b,c, and attempts to reach the goal near the distant yellow area at (3,0).

### 4.3 Model setups

To evaluate the impact of our proposed reward terms on collision avoidance, goal reaching, and sim-to-real generalization performance, we design five (5) models for comparative experiments. The first model, $W_{\text{obst}}$ , serves as the baseline and is implemented using the TD3 algorithm with baseline reward terms and $R_{\text{obst}}$ . This model is a basic version that can be trained to convergence in the simulation environment.

The second model, $W_{\text{C}}$ , is designed to test the effect of the $R_{\text{Coulomb}}$ term, with a reward function comprising the baseline and $R_{\text{Coulomb}}$ . Ideally, due to the repulsive and attractive forces, $W_{\text{C}}$ should achieve a higher obstacle-avoidance success rate and reach the goal more efficiently compared to $W_{\text{obst}}$ .

The third model, $W_{\text{C}+\text{v}}$ , is designed to test whether the combination of $R_{\text{Coulomb}}$ and $R_{\text{vision}}$ further improves the success rates of obstacle avoidance. From a design perspective, we expect that this reward combination to yield a more robust robot maneuver ability, with a higher success rate than the previous two models.

The fourth model, $W_{\text{C}+\text{obst}}$ , incorporates the baseline reward terms along with $R_{\text{obst}}$ and $R_{\text{Coulomb}}$ . We expect this model to perform slightly better than $W_{\text{C}}$ , but not as well as $W_{\text{C}+\text{v}}$ . The fifth model, $W_{\text{C}+\text{v}+\text{obst}}$ , includes all the reward terms in Equation 2. Since our proposed reward terms, $R_{\text{Coulomb}}$ and $R_{\text{vision}}$ are designed to enhance the robot’s collision-avoidance ability, we expect this model outperforms $W_{\text{obst}}$ , $W_{\text{C}}$ and $W_{\text{C}+\text{obst}}$ , while achieving performance comparable to $W_{\text{C}+\text{v}}$ .

These five models are constructed through different combinations of reward terms. By comparing models with and without the $R_{\text{Coulomb}}$ term, we can evaluate its contribution to collision-free motion planning. Similarly, by comparing models with and without the $R_{\text{vision}}$ term, we can evaluate the role of $R_{\text{vision}}$ in robot maneuver. Using this controlled-variable approach, we can systematically test and validate the effectiveness of the proposed reward terms, $R_{\text{Coulomb}}$ and $R_{\text{vision}}$ .

### 4.4 Evaluation metrics

To more comprehensively assess the navigation and collision avoidance performance of different models, three quantitative metrics were employed: Success Rate (SR), Collision Ratio (CR), and Average Goal Distance (GD). These metrics jointly evaluate navigation reliability, safety, and efficiency, enabling a more rigorous comparison among all tested models and environments. The details of these three metrics are described below:

- SR: Success rate is defined as the ratio of successful rollouts, where the robot reaches the goal without collision, to the total number of rollouts. It measures the overall reliability of the navigation policy in completing tasks successfully. A higher SR indicates stronger obstacle avoidance and goal-reaching capability. SR serves as the primary evaluation metric in both simulation and real-world experiments.

$$SR=\frac{\text{success\_count}}{\text{rollout}}$$

- CR: Collision ratio is designed to evaluate navigation safety and efficiency when the total runtime cannot be directly compared. In our simulation, each episode restarts from the initial position after collision, and start-goal pairs differ across different model tests, making navigation time or path length unsuitable for fair comparison. Therefore, CR reflects the frequency of collisions normalized by total steps, indicating how safely and efficiently the robot navigates over longer trajectories. A lower CR means the model can operate longer with fewer collisions, demonstrating better collision-avoidance capability and stability. CR is evaluated only in simulation experiments.

$$CR=\frac{\text{collision\_count}}{\text{total\_steps}/1000}$$

- Avg. GD: Average goal distance captures the model’s tendency to approach the goal, even in failed attempts. While SR only measures how often a model reaches the goal, GD quantifies how close the robot remains to the target at the end of each rollout. A lower GD indicates that the model either successfully reaches the goal or, in failure cases, terminates nearer to it, which demonstrates stronger goal-reaching tendency and better awareness of feasible solutions. This metric is evaluated only in simulation for controlled quantitative comparison.

$$Avg.GD=\frac{\text{total\_goal\_distance}}{\text{rollout}}$$

## 5 Experiments and results

The major innovation of our design lies in the two reward components, $R_{\text{Coulomb}}$ and $R_{\text{vision}}$ , in Equation 2. We conducted a series of experiments to evaluate their effectiveness through statistical analysis and visual inspection. The models are tested and compared in both simulation and real-world environments.

### 5.1 Results in simulation environments

**FIGURE 10:**
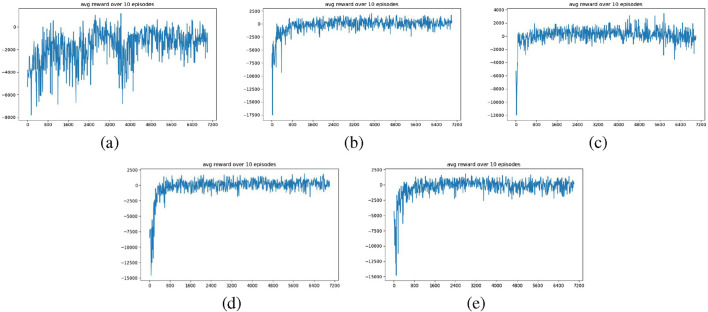
Plots of training results (epoch = 7,000) for models: **(a)** $W_{\text{obst}}$ , **(b)** $W_{\text{C}}$ , **(c)** $W_{\text{C}+\text{v}}$ , **(d)** $W_{\text{C}+\text{obst}}$ and **(e)** $W_{\text{C}+\text{v}+\text{obst}}$ .

The five (5) models are trained in the training environment for 7,000 epochs, and the corresponding training data are shown in Figure 10. The figure plots the average reward obtained over each set of 10 epochs against the training epochs. From Figure 10, we observe that $W_{\text{obst}}$ converges the slowest, stabilizing only after approximately 4,800 epochs, and its performance between 4,800 and 7,000 epochs remains less consistent than that of the other four models. $W_{\text{C}}$ and $W_{\text{C}+\text{obst}}$ converge much faster, beginning around 800 epochs, with stable performance after convergence. $W_{\text{C}+\text{v}}$ and $W_{\text{C}+\text{v}+\text{obst}}$ converges even faster, showing rapid convergence around 400 epochs. These results suggest that the $R_{\text{Coulomb}}$ term significantly enhances training efficiency, while the $R_{\text{vision}}$ term further accelerates convergence.

After training converged, the models were evaluated in the test scenes using success rate as the performance metric. Table 1 summarizes the results of the five trained models tested across four (4) simulation environments. Dynamic indicates the presence of moving obstacles, while unseen refers to deployment in a previously unseen (new) environment.

**TABLE 1:** Quantitative test results for $W_{\text{obst}}$ , $W_{\text{C}}$ , $W_{\text{C}+\text{v}}$ , $W_{\text{C}+\text{obst}}$ and $W_{\text{C}+\text{v}+\text{obst}}$ in simulation environments. Metrics: SR (in %), CR and Avg. GD (in meters). Rollout = 200.

Test scene	Metric	$W_{\text{obst}}$	$W_{\text{C}}$	$W_{\text{C}+\text{v}}$	$W_{\text{C}+\text{obst}}$	$W_{\text{C}+\text{v}+\text{obst}}$
Test 0 (static)	SR (%)	71.00	90.00	96.00	96.00	**97.50**
	CR	0.256	0.069	0.029	0.028	**0.017**
	Avg. GD (m)	0.639	0.166	0.077	0.056	**0.031**
Test 1 (dynamic)	SR (%)	59.50	73.00	82.00	82.50	**83.00**
	CR	0.396	0.220	0.143	0.146	**0.129**
	Avg. GD (m)	0.740	0.425	0.344	**0.305**	0.332
Test 2 (static)	SR (%)	59.00	85.00	86.00	87.00	**87.50**
	CR	0.366	0.105	0.100	0.262	**0.068**
	Avg. GD (m)	0.850	0.295	**0.203**	0.261	0.277
Test 3 (dynamic)	SR (%)	44.50	59.50	61.50	62.00	**69.50**
	CR	0.567	0.288	0.257	0.239	**0.189**
	Avg. GD (m)	0.776	0.557	0.593	0.549	**0.546**

Bold values indicate the best performance for each metric.

The key observations are as follows:

1. The $W_{\text{obst}}$ model performs the worst across all test environments, exhibiting the lowest success rate, the highest collision ratio and the highest average goal distance. This confirms its limited effectiveness when used alone.
2. Incorporating Coulomb reward components $\left(R_{\text{Coulomb}}\right)$ consistently boosts robot performance across all test scenes with higher success rate, lower collision ratio and lower average goal distance, validating the effectiveness of our reward design.
3. Adding vision rewards $\left(R_{\text{vision}}\right)$ further improves success rate and reduces collision ratio and average goal distance, as seen in the comparisons of $W_{\text{C}}$ vs. $W_{\text{C}+\text{v}}$ and $W_{\text{C}+\text{obst}}$ vs. $W_{\text{C}+\text{v}+\text{obst}}$ .
4. The overall best-performing model in simulation, based on success rate, collision ratio and average goal distance is $W_{\text{C}+\text{v}+\text{obst}}$ .

### 5.2 Evaluation of robot performance in real environments

To evaluate the trained policies on a real robot, we deployed the five DRL models onto a TB3. As described in the previous subsection, the test environments are categorized into three types: Real Test Scene 1, Scene 2, and Scene 3. The robot trajectorieshe test environments are categorized into three typ are visualized in RViz using green dots, which represent real-time TB3 position data.

**FIGURE 11:**
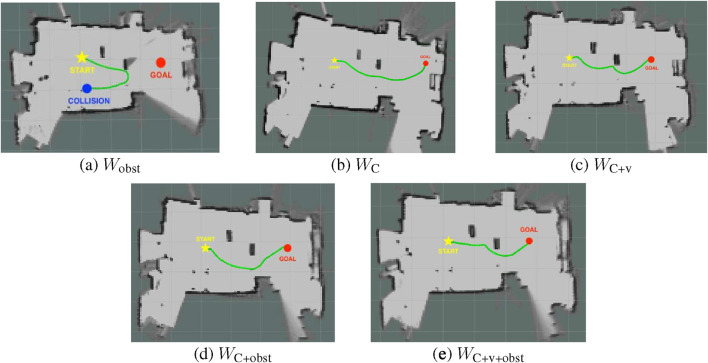
Plots of trajectories for the models in Real Test Scene 1 with static obstacles. **(a)** $W_{\text{obst}}$ failed, **(b)** $W_{\text{C}}$ succeeded, **(c)** $W_{\text{C}+\text{v}}$ succeeded, **(d)** $W_{\text{C}+\text{obst}}$ succeeded and **(e)** $W_{\text{C}+\text{v}+\text{obst}}$ succeeded. In each test, the start position is marked by a yellow star, the goal position by a red dot, and collisions by blue dots.

The setups and results are as follows. In Real Test Scene 1, two static obstacles are placed directly between the start point and the goal, requiring the TB3 to navigate around them to reach its destination. This setup evaluates each model’s collision-avoidance capability under the sim-to-real challenge. From the trajectories shown in Figure 11, we observe that although $W_{\text{obst}}$ initially moves toward the goal, it fails to find a feasible path after attempting to avoid an obstacle and ultimately collides with a table leg.

In contrast, the other four models successfully navigate around the obstacles and reach the goal. Their trajectories in this test scene are generally similar, all bypassing the obstacles from the right side with slight variations in clearance. Among them, $W_{\text{C}+\text{obst}}$ maintained the greatest distance from obstacles during navigation. This behavior can be attributed to the influence of the $R_{\text{obst}}$ and $R_{\text{repu}}$ terms in the reward function, which encouraged the TB3 to maintain a larger buffer from obstacles.

**FIGURE 12:**
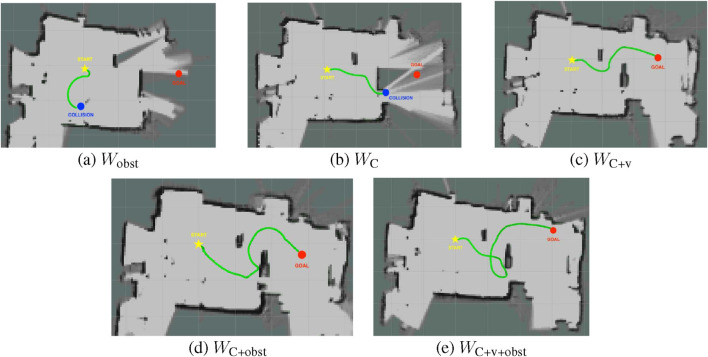
Plots of trajectories for models under Real Test Scene 2 with static obstacles. **(a)** $W_{\text{obst}}$ failed, **(b)** $W_{\text{C}}$ failed, **(c)** $W_{\text{C}+\text{v}}$ succeeded, **(d)** $W_{\text{C}+\text{obst}}$ succeeded and **(e)** $W_{\text{C}+\text{v}+\text{obst}}$ succeeded.

In Real Test Scene 2, to further challenge the models, we extended the second obstacle to evaluate their ability to find an alternative path when the previous route was no longer feasible. Figure 12 illustrates the trajectory results of the five models. It can be observed that $W_{\text{obst}}$ and $W_{\text{C}}$ failed to reach the goal: $W_{\text{obst}}$ collided with a table leg after moving toward the goal, while $W_{\text{C}}$ followed its previous path and crashed into the extended obstacle. In contrast, $W_{\text{C}+\text{v}}$ , $W_{\text{C}+\text{obst}}$ , and $W_{\text{C}+\text{v}+\text{obst}}$ all successfully reached the goal.

Among the successful models, $W_{\text{C}+\text{v}}$ demonstrated the most reasonable trajectory, maintaining the largest collision-free buffer. The effect of the vision reward $\left(R_{\text{vision}}\right)$ can be observed by comparing $W_{\text{C}+\text{v}}$ (Figure 12c) with models that do not incorporate vision (Figures 12a,b,d). Vision reward, based on LiDAR segmentation, enables the robot to detect obstacles from a distance, leading to earlier avoidance and smoother trajectories.

**FIGURE 13:**
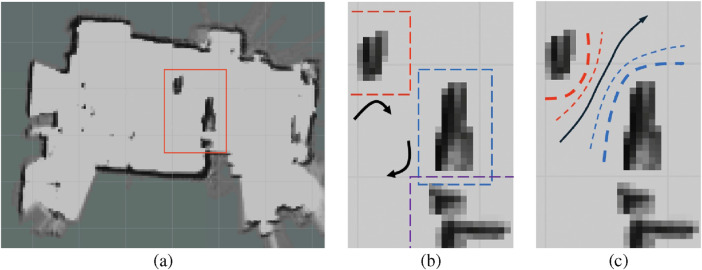
Environment overview with a red bounding box in **(a)** indicating the zoom-in region, and detailed views of the highlighted area in **(b,c)**. In **(b,c)**, the black arrowed curves show the robot’s motion under obstacle influence with $R_{\text{obst}}$ and $R_{\text{Coulomb}}$ respectively.

The effectiveness of the Coulomb reward $\left(R_{\text{Coulomb}}\right)$ compared to the obstacle reward $\left(R_{\text{obst}}\right)$ in Test Scene 2 is illustrated in Figures 13. Figures 13b,c show a zoomed-in region of the scene (highlighted by the red box in Figure 13a). As shown in Figure 13b, the obstacle reward essentially creates rigid surrounding zones that the robot cannot penetrate. Consequently, the robot is repeatedly repelled, first from the upper red-box region and then from the blue-box region. This sequence of rejections prevents the robot from finding a feasible path between the two obstacles. In contrast, the Coulomb reward, $R_{\text{Coulomb}}$ , varies smoothly and provides global guidance across the entire field. While still discouraging the robot from moving too close to obstacles, it forms a watershed-like potential field that directs the robot toward the goal more effectively. This mechanism partly explains the superior performance of $W_{\text{C}+\text{v}}$ , as shown in Figure 12c.

In Real Test Scene 3, moving obstacles were placed along the TB3’s travel path to evaluate its ability to avoid obstacles in dynamic environments. Based on visual inspection, $W_{\text{C}+\text{v}}$ performed best, consistently avoiding obstacles and reaching the goal with relatively short paths. The second-best model was $W_{\text{C}+\text{v}+\text{obst}}$ , which also avoided obstacles and reached the goal with high probability. For demonstration, see the accompanying YouTube video.

We conducted a statistical analysis of the five models across the three real-world test scenes. Each model was run five times (i.e., Epoch = 5), and the average success rates are summarized in Table 2. These results confirm our visual observations: $W_{\text{C}+\text{v}}$ achieved the best overall performance across all scenes, followed by $W_{\text{C}+\text{v}+\text{obst}}$ . However, this finding contrasts with the simulation results, where $W_{\text{C}+\text{v}+\text{obst}}$ showed the best performance.

**TABLE 2:** Statistical results for $W_{\text{obst}}$ , $W_{C}$ , $W_{\text{C}+\text{v}}$ , $W_{\text{C}+\text{obst}}$ , and $W_{\text{C}+\text{v}+\text{obst}}$ under real-world test scenes. SR represents for Success Rate (%). Rollout = 5.

Test scene	Metric	$W_{\text{obst}}$	$W_{\text{C}}$	$W_{\text{C}+\text{v}}$	$W_{\text{C}+\text{obst}}$	$W_{\text{C}+\text{v}+\text{obst}}$
Test scene 1 (static)	SR (%)	0	80.00	**100.00**	60.00	**100.00**
Test scene 2 (static, wider obj.)	SR (%)	0	0	**80.00**	20.00	60.00
Test scene 3 (dynamic)	SR (%)	0	0	**100.00**	40.00	80.00

Bold values indicate the highest success rate (SR) under each test scene.

**FIGURE 14:**
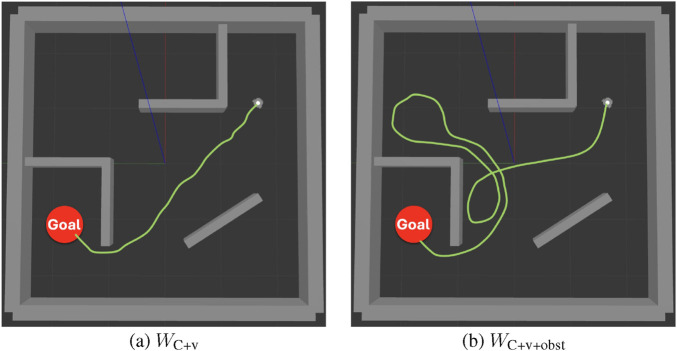
Schematic path comparison under the same start and goal: **(a)** $W_{\text{C}+\text{v}}$ , **(b)** $W_{\text{C}+\text{v}+\text{obst}}$ . Green curves indicate illustrative trajectories only and are not directly generated by the robot.

After examining the actual trajectories taken by the robots in simulation tests, we identified a plausible explanation, illustrated in Figure 14. As shown in the figure, under identical start and goal positions, $W_{\text{C}+\text{v}+\text{obst}}$ tended to generate more detours. While such behavior had little impact in idealized simulations, it posed challenges in real-world deployment. Increased detours elevated the risk of failure due to collisions, loss of the goal, or becoming stuck, ultimately contributing to the lower success rate observed for $W_{\text{C}+\text{v}+\text{obst}}$ in real-world tests. Our findings are consistent with those of Da et al. (Da et al., 2025), who noted that the sim-to-real gap arises primarily from perception-related limitations and execution discrepancies.

To summarize, TB robots equipped with the trained motion planning policies ( $W_{\text{C}+\text{v}}$ and $W_{\text{C}+\text{v}+\text{obst}}$ ) exhibit strong maneuvering capabilities in complex simulation and real environments, demonstrating the effectiveness of our design.

### 5.3 Additional cluttered simulated environments and test results

**FIGURE 15:**
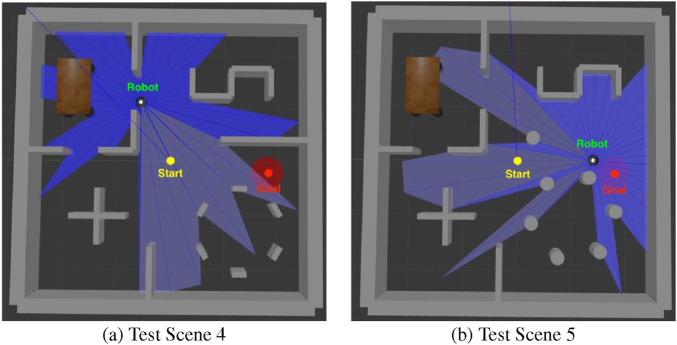
Additional cluttered environments introduced in response to the concerns. **(a)** test scene 4 includes static obstacles only, **(b)** test scene 5 introduces additional moving obstacles, which are cylindrical in shape.

To further validate the robustness and generalization of the proposed DRL framework, two additional cluttered simulation environments were constructed, as shown in Figure 15.

Test Scene 4 (illustrated in Figure 15a) contains densely arranged static obstacles, forming multiple narrow corridors and enclosed regions that challenge precise local navigation and obstacle avoidance. Test Scene 5 (shown in Figure 15b) extends this design by introducing multiple cylindrical obstacles that move dynamically along predefined trajectories. As these cylindrical obstacles move, the layout of traversable space changes over time, forming a dense and dynamic scene that explicitly requires adaptive path planning.

Both environments substantially increase the navigation difficulty compared with the Test Scenes 0–3, imposing tighter spatial constraints and more complex interactions between the robot, obstacles, and goal. The policies trained in earlier experiments were directly deployed in these new scenes without retraining to evaluate cross-environment generalization.

The quantitative outcomes summarized in Table 3 provide a detailed characterization of policy robustness as environmental complexity increases from the previously tested sparse settings (Test Scenes 0–3) to the newly introduced cluttered environments (Test Scenes 4–5). Across all five (5) models, performance declined notably as the environment became more cluttered. This drop was primarily caused by the reduced navigable space and the increased probability of the agent becoming trapped in local minima created by narrow corridors and complex obstacle layouts.

**TABLE 3:** Quantitative results in additional cluttered environments. SR represents for Success Rate (%), CR denotes Collision Rates, and Avg. GD (m) is the averaged distance from robot to goal at the end of each run, measured in meters. Rollout = 200.

Test scene	Metric	$W_{\text{obst}}$	$W_{\text{C}}$	$W_{\text{C}+\text{v}}$	$W_{\text{C}+\text{obst}}$	$W_{\text{C}+\text{v}+\text{obst}}$
Test scene 4 (static)	SR (%)	49.00	63.50	**79.00**	73.50	63.00
	CR	0.606	0.359	**0.208**	0.269	0.373
	Avg. GD (m)	0.868	0.709	**0.387**	0.518	0.730
Test scene 5 (dynamic)	SR (%)	39.00	39.00	**62.00**	57.00	43.00
	CR	0.93	0.76	**0.39**	0.47	0.68
	Avg. GD (m)	1.087	0.855	**0.629**	0.736	1.016

Bold values indicate the best performance for each metric.

Nevertheless, the Coulomb- and vision-guided model $W_{\text{C}+\text{v}}$ consistently achieved the highest success rates and the lowest collision ratios and average goal distance in both cluttered settings. In Test 5 (dynamic clutter), moving obstacles further lowered the success rate for all methods, but $W_{\text{C}+\text{v}}$ remained the most robust. This superior performance confirms that the Coulomb-based reward, which effectively models obstacle and goal forces, provides a strong global guidance signal. Furthermore, the inclusion of the vision input further improves performance by giving the agent richer spatial information and helping it adapt when the free space changes over time. The low CR values reflect the model’s superior collision-avoidance capability, while the low average GD suggests that $W_{\text{C}+\text{v}}$ either successfully reached the goal or, in failure cases, terminated closer to the goal than other models.

When compared to the earlier, sparser Test Scene 0–3 environments, the benefit of using Coulomb- and vision-guided policy becomes more obvious as the environment gets more cluttered, while purely using $R_{\text{obst}}$ model’s performance drops quickly. This trend aligns with the real-world outcomes discussed previously: $W_{\text{C}+\text{obst}+\text{v}}$ showed degraded performance in the physical setup because the environment is more cluttered, and the $R_{\text{obst}}$ reward can dominate in narrow passages, effectively blocking feasible routes. The new cluttered-scene results further confirm that $W_{\text{C}+\text{v}}$ is the most effective model, showing strong path adaptability and efficient route generation even in dense or dynamic environments.

## 6 Conclusion

In this paper, we presented a physics-inspired DRL framework for mobile robot motion planning that leverages Coulomb-force modeling to provide interpretable and effective guidance. By representing the robot, goal, and obstacles as electrical charges, we introduced a novel Coulomb-based reward mechanism that delivers smooth, pervasive, and consistent signals during training. To the best of our knowledge, this is the first work to employ Coulomb forces in path planning and reinforcement learning.

Our approach further incorporates obstacle boundaries extracted from LiDAR segmentation, enabling the robot to anticipate and avoid collisions in advance. Through training in a digital twin environment and deployment on a real TB3 robot, we demonstrated that the proposed framework significantly reduces collisions, maintains safe obstacle clearances, and improves trajectory smoothness across both simulated and real-world scenarios. These results confirm not only the effectiveness but also the strong explainability of our Coulomb- and vision-based rewards in shaping robot behavior.

Finally, by carefully designing environment-invariant components, our system exhibits enhanced generalization, suggesting broad applicability to diverse navigation tasks. Moving forward, this framework provides a promising foundation for extending physics-inspired reinforcement learning to multi-robot systems, more complex environments, and real-time adaptive planning.

## Data Availability

The raw data supporting the conclusions of this article will be made available by the authors, without undue reservation.
